# Characterization of a Novel Cotton Subtilase Gene *GbSBT1* in Response to Extracellular Stimulations and Its Role in Verticillium Resistance

**DOI:** 10.1371/journal.pone.0153988

**Published:** 2016-04-18

**Authors:** Xingpeng Duan, Zhidong Zhang, Jin Wang, Kaijing Zuo

**Affiliations:** 1 Plant Biotechnology Research Center, School of Agriculture and Life Sciences, Shanghai Jiao Tong University, Shanghai, China; 2 Biotechnology Research Institute, Chinese Academy of Agricultural Sciences, Beijing, China; National Key Laboratory of Crop Genetic Improvement, CHINA

## Abstract

Verticillium wilt is a disastrous vascular disease in plants caused by *Verticillium dahliae*. *Verticillium* pathogens secrete various disease-causing effectors in cotton. This study identified a subtilase gene *GbSBT1* from *Gossypium babardense* and investigated the roles against *V*. *dahliae* infection. *GbSBT1* gene expression is responsive to *V*. *dahliae* defense signals, jasmonic acid, and ethylene treatments. Moreover, the GbSBT1 protein is mainly localized in the cell membrane and moves into the cytoplasm following jasmonic acid and ethylene treatments. Silencing *GbSBT1* gene expression through virus-induced *GbSBT1* gene silencing reduced the tolerance of Pima-90 (resistant genotype), but not facilitated the infection process of *V*. *dahliae* in Coker-312 (sensitive genotype). Moreover, the ectopically expressed *GbSBT1* gene enhanced the resistance of *Arabidopsis* to *Fusarium oxysporum* and *V*. *dahliae* infection and activated the expression levels of defense-related genes. Furthermore, pull-down, yeast two-hybrid assay, and BiFC analysis revealed that GbSBT1 interacts with a prohibitin (PHB)-like protein expressed in *V*. *dahliae* pathogens during infection. In summary, GbSBT1 recognizes the effector PHB protein secreted from *V*. *dahliae* and is involved in *Verticillium*-induced resistance in cotton.

## Introduction

Verticillium wilt, a devastating disease of more than 200 crops worldwide, is typically caused by the soil-borne fungus *Verticillium dahliae* [1]. *V*. *dahliae* secretes various effectors to evade the guard system or destroy the innate immune system of host plants [2,3,4,5]. The *V*. *dahliae* genome encodes approximately 780 secreted proteins containing signal peptides as candidate effectors [6]. These secreted proteins, including diverse polysaccharide lyases, could cleave different forms of pectins in host plants to help Verticillium wilt-causing pathogens invade xylem vessels [3,7]. *V*. *dahliae* also secretes isochorismatases (without signal peptide) that suppress salicylate-mediated innate immunity in host plants [4]. In the absence of its corresponding R protein (Ve1), the *Verticillium* effector Ave1 functions in the apoplast of host plants to promote pathogenicity [3]. Nevertheless, the mechanism by which these effectors from *Verticillium* pathogens are recognized or primed by host plants remains largely unknown.

Subtilisin-like proteases (subtilase) are extracellular and broad-spectrum serine proteases containing a catalytic triad motif that consists of aspartate, histidine, and serine [8]. The subtilase gene family in *Arabidopsis* is composed of 56 members classified into six distinct subfamilies [9,10]. Recent studies have revealed that subtilase genes are specifically induced following pathogenic infection and are hypothesized to be involved in pathogen recognition and immune priming. SBT3.3 rapidly responds to pathogenic infection and activates innate immunity preceding the activation of SA responsive genes [11]. P69, the first identified plant subtilase to be identified located in the vacuole and intercellular space, is specifically responsible for the *in vivo* pathogenesis-associated processing of LRP (a leucine-rich repeat protein) [12,13]. Furthermore, AtSBT1.1 specifically cleaves proAtPSK4 into the mature peptide growth factor AtPSK4 to promote callus formation in culture and fungal resistance in *Arabidopsis* [14]. Combined data indicate that subtilases function as catalytic proteases to recognize pathogenic attacks [10].

This study characterized an extracellular subtilase gene (*GbSBT1*) from the *Gossypium barbadense* variety Pima-90. *GbSBT1* knockdown reduced the defenses of *G*. *barbadense* against *V*. *dahliae* attack, and the cotton plants exhibited a more severe wilting phenotype than the control plants. Ectopically expressed *GbSBT1* gene enhanced the disease tolerance of *Arabidopsis* against *F*. *oxysporum* and *V*. *dahliae*. Importantly, GbSBT1 interacted with the protein prohibitin (PHB) secreted by *V*. *dahliae*. Our results proved that GbSBT1 functions as a sensing protein during *V*. *dahliae* infection and activates downstream resistance response in cotton.

## Materials and Methods

### Plant materials and pathogen culture

Cotton seeds (*G*. *barbadense* variety Pima-90 and *Gossypium hirsutum* variety Coker-312) were soaked in corrosive sublimate (1/1000, v/v) for 5 min and then washed three times with sterile water. The aseptic seeds were grown in MS medium, and 1-week-old cotton seedlings were used in subsequent experiments. Wild-type (WT; ecotype Columbia, Col-0) and transgenic *Arabidopsis* plants were grown in a greenhouse under long-day conditions (22°C, 16/8 h light/dark).

The defoliating isolate V991 of *V*. *dahliae* was grown on Czapek’s medium agar medium (NaNO_3_, 0.3% w/v; MgSO_4_, 0.1% w/v; KH_2_PO_4_, 0.1% w/v; FeSO_4_, 0.0002% w/v; KCl, 0.1% w/v; sucrose, 3% w/v; and agar 3% w/v; pH 6.0) for 7 days [15]. Conidial spores were harvested and adjusted to 1 × 10^6^ spores mL^−1^ with sterile distilled water. For *F*. *oxysporum*, the isolate was inoculated on Bilai’s medium plates. After 4 days, conidial spores were harvested and adjusted to 1 × 10^6^ spores mL^−1^ with sterile distilled water. *V*. *dahliae* and *F*. *oxysporum* spore suspensions were then used in the inoculation experiments in cotton and *Arabidopsis*, respectively.

### *GbSBT1* gene isolation from sea-island cotton

To clone the subtilase gene from sea-island cotton, first-strand cDNA was synthesized from 1 μg of total RNA by using PrimeScript RT reagents and a gDNA Eraser kit (TaKaRa, Japan). The synthesized first-strand cDNA served as the template in reverse transcript polymerase chain reaction (RT-PCR). Gene-specific primers (S1 Table) were used to amplify the full-length subtilase gene. The PCR reactions (25 μL) contained 10 ng first-strand cDNA, 1 U ExTaq, 10 pM dNTPs, 5 pM MgCl_2_, and 10 pM primers. RT-PCR was performed under the following conditions: initial reaction at 94°C for 5 min, followed by 30 cycles at 94°C for 30 s, 54°C for 30 s, 72°C for 2 min and 30 s, and 72°C for 10 min. The amplified gene was cloned into the *pGEM-T* vector (Clontech, USA) and then confirmed by DNA sequencing. The cloned subtilase gene was named *GbSBT1*.

The conservative domains of GbSBT1 (the signal peptide, the putative inhibitor domain, and the putative catalytic sites) were searched online through the domain Blast (www.ncbi.nlm.nih.gov). We aligned GbSBT1 with the subtilase proteins of three organisms by using Bioedit to analyze the relationship of GbSBT1 with other subtilases. A neighbor-joining tree of different subtilases was constructed using MEGA 3.0 [16].

### Expression pattern analysis of the *GbSBT1* gene

Cotton seeds were soaked in corrosive sublimate (1/1000, v/v) for 5 min and then washed three times with sterile water. The aseptic seeds were grown in MS medium at the appropriate condition (28±2°C, 14/10 h light/dark), and 2-week-old cotton seedlings were used in subsequent experiments. Sterilized young cotton seedlings were placed in V991 suspension liquid (1 × 10^6^ conidia mL^−1^). Cotton roots were harvested at 2, 4, 6, 8, 12, 24, 36, and 48 h after inoculation. Cotton roots inoculated with 1/2MS medium without mycelia served as the control. Total RNA was extracted using RNA prep Pure Plant Kit (DP441; Tiangen, China) in accordance with the manufacturer’s instructions.

The expression pattern of the *GbSBT1* gene was analyzed using qRT-PCR in accordance with the manual of SYBR premix Ex-Taq (Takara, Japan) in a DNA Engine Option 3 System (MJ Research, USA). The ubiquitin gene was amplified using the primers as endogenous controls. Changes in gene expression were calculated using the comparative ΔCT method. Each sample was repeated at least thrice, and the amplification results were analyzed using the Option 3 software. The primers used in qRT-PCR are listed in S1 Table.

### Subcellular localization of GbSBT1 protein

To investigate the subcellular localization of the GbSBT1 protein, we cloned the coding region of the *GbSBT1* gene and its ORF without signal peptide-encoding nucleotides into the *pEG101-YFP* vector to generate *pEG101-*(*CaMV35S*::*GbSBT1* or *GbSBT1*△*SP-YFP*::*NOS*) constructs. The recombinant plasmids were transformed into the *Agrobacterium* strain EHA105. Nearly fully expanded 3-week-old tobacco (*Nicotiana benthamiana*) leaves were infiltrated with the *Agrobacterium* that was diluted into OD600 of 0.6–0.8 with the solution (10 mM MES, pH 5.6; 10 mM MgCl_2_, and 150 mM acetosyringone). After 2–4 days, the fluorescence signals in infiltrated leaves were analyzed through confocal microscopy (Leica TCS SP5).

To determine whether or not GbSBT1 is localized on the cell membrane, we co-expressed the GbSBT1-GFP fusion protein with plasma membrane integral protein PIP1-mCherry (cell membrane-localized) in tobacco leaf epidermal cells according to the above-mentioned method. Three days after infiltration, the tobacco leaves were cut into strips and then digested into the protoplasts according to the reported method [17]. Confocal images of tobacco protoplasts expressing both GbSBT1-GFP and plasma membrane integral protein PIP1-mCherry proteins were observed.

### Virus-induced *GbSBT1* gene silencing (VIGS) in cotton and *V*. *dahliae* resistance analysis

VIGS was used to investigate the function of the *SBT1* gene in resistant (Pima-90) and sensitive genotypes (Coker-312) as previously described method [18,19]. *GbSBT1* and *GhSBT1* gene-specific fragments (corresponding to the 225^th^-448^th^ amino acids (aa) in the GbSBT1 protein and not detected in other genes at the genomic level) were amplified using gene-specific primers (S1 Table) and then inserted into the *pDONR201* vector. The *GbSBT1*-specific fragment was subsequently recombined with the *pYL156* vector. Plasmids containing the binary TRV vectors *pTRV-RNA1*, *pTRV-RNA2*, and *pYL156* derivatives were transformed into *Agrobacterium* strain GV3101. *Agrobacterium* strains with different derivatives were grown in LB medium containing 50 μg mL^−1^ kanamycin, 50 μg mL^−1^ rifampicin, 10 mM MES, and 200 μM acetosyringone. The *Agrobacterium* cultures were harvested by centrifugation, resuspended in a solution (10 mM MES and 200 μM MgCl_2_), and then incubated for at least 3 h at room temperature.

The *Agrobacterium* harboring *pTRV-RNA1* was mixed with the *Agrobacterium* harboring *pTRV-RNA2* and *pTRV-GbSBT1* or *pTRV-GhSBT1* at a 1:1 ratio. A needle-free injection syringe was used to inject the mixed *Agrobacterium* cultures into the cotyledons of 2-week-old cotton plants. The injected cotton plants were grown in a greenhouse for 2 weeks to repress *GbSBT1* or *GhSBT1* gene expression.

Conidial suspensions of *V*. *dahliae* isolate V991 (1 × 10^6^ spores mL^−1^) were stem-inoculated into both control and VIGS plants by using a syringe needle approximately 1 cm below the cotyledons [19]. After 7 days, the disease symptom, which was divided into 5 levels (level 0–4) on the basis of severity, was investigated in the cotton seedlings. The plant disease indexes (DI) were then calculated as follows: DI = (∑(*n* × number of seedlings at level n))/(4 × number of total seedlings × 100), where n denotes the severity of the disease level of the cotton seedlings [19]. VIGS experiments were repeated at least thrice using more than 10 cotton plants for each constructs.

To analyze *V*. *dahliae* infection in the control and VIGS, diaminobenzidine (DAB) staining of cotton leaves was performed as previously described [19]. The DAB staining experiments were repeated at least thrice.

### Generation of transgenic *GbSBT1 Arabidopsis* plants and pathogen resistance analysis

Wild-type *Arabidopsis thaliana* plants (ecotype Columbia, Col-0) were grown in the greenhouse under long-day conditions (22°C, 16/8 h light/dark) according to Huang et al. [20]. We cloned the coding region of the *GbSBT1* gene into the *pDONR201* vector to generate the *pDONR-GbSBT1* construct. The *GbSBT1* gene was then recombined into the *pEG101* vector via the Gateway LR recombination reaction (Invitrogen, CA, USA) to generate the *pEG101-35S*::*GbSBT1*::*NOS* expression cassette. The construct was transferred into *Agrobacterium tumefaciens* GV3101 and then introduced into *Arabidopsis* (ecotype Columbia) plants using the floral dip method [21]. Fully mature seeds were collected and screened by 20 mg L^−1^ BSATA. Herbicide-resistant plants were confirmed via PCR and self-crossed to generate homogenous T3 lines.

The WT and transgenic lines were grown in a greenhouse for 2 weeks and then used for *F*. *oxysporum* and *V*. *dahliae* resistance analysis. The leaves of the WT and transgenic lines were inoculated with 10 μL conidial suspensions (1 × 10^7^ conidia ml^−1^) of *F*. *oxysporum* and *V*. *dahliae*. Distilled water with 0.2% Tween-20 without conidia served as the control. The severity of the disease in the WT and transgenic *GbSBT1* lines was assessed after a week. At least three biological replicates were performed in the *F*. *oxysporum* and *V*. *dahliae* resistance analysis.

### Expression analysis of defense-related genes and trypan blue staining of *Arabidopsis* leaves followed by pathogen inoculation

qRT-PCR was used to analyze the expression patterns of defense-related genes in WT and transgenic *Arabidopsis* plants after pathogen inoculation. Leaves of the WT and transgenic *Arabidopsis* plants were collected for RNA isolation in different intervals after *F*. *oxysporum* inoculation. qRT-PCR analysis was performed as described above [20]. The primers used in qRT-PCR are listed in S1 Table, and in *Arabidopsis*, *Actin 2* was chosen as the control. Each sample was repeated at least thrice, and the amplification results were analyzed using the Option 3 software.

Conidial suspensions of *F*. *oxysporum* were inoculated on leaves of 4-week-old WT and transgenic *GbSBT1 Arabidopsis* lines for 2 days. To determine pathogen colonization, the inoculated leaves were stained with trypan blue [19]. The leaves were boiled in a lactophenol-trypan blue solution (10 mL lactic acid, 10 mL glycerol, 10 mg phenol, and 10 mg trypan blue dissolved in 10 mL distilled water) for 2 min and then cleared with chloral hydrate (2.5 g mL^−1^) overnight. The trypan blue staining experiments were repeated at least thrice.

### Identification of GbSBT1-interacting proteins through pull-down assay

The *Agrobacterium* strain *GV3101* containing the *pEG101-35S*::*GbSBT1-GFP*::*Nos* plasmid and the control plasmid were transformed into cotton cotyledons for the GbSBT1-GFP fusion protein or GFP expression (control). After 12 h, *V*. *dahliae* V991 was inoculated into the cotyledons of Coker-312. The total proteins of the cotton cotyledons expressing the GbSBT1-GFP fusion protein or the GFP protein were extracted after 24 h for pull-down assay. This assay was performed in accordance with the protocol of FOCUS Plant Proteome (Sangon, China), which is briefly described below.

The GFP monoclonal antibody was covalently linked to Sepharose beads and washed twice with washing buffer. The extracted total protein of the cotton cotyledons inoculated with V991 was loaded into the column and then incubated with the GFP antibody beads. After 30 min of incubation, the Sepharose beads combined with GFP antibody-cotton proteins were washed thrice. Finally, GbSBT1-interacting candidates were eluted with the lysis buffer and then collected in the loading buffer.

The eluted proteins were confirmed via SDS-PAGE. The GbSBT1-interacting proteins and the control (GFP-binding) were identified using Nano-Liquid Chromatography (UltiMate3000 RSLCnano Liquid Chromatography, Bruker Daltonics) and Quadrupole-Time-of-Flight Mass Spectrometer (maXis impact UHR-TOF MS, Thermofisher). The sequenced peptides were mapped to the *V*. *dahliae* genome and annotated by the hits. The GbSBT1-interacting proteins without background noise were then analyzed further.

### Yeast two-hybrid assay and BiFC confirmation *in vivo*

To test protein interaction *in vitro*, we cloned the genes encoding the benzodiazepine receptor family protein (Accession NO: XP_009649206), peptidyl-tRNA hydrolase (Accession NO: XP_009656275), PHB (Accession NO: XP_009649890), and *GbSBT1* into both *pGBKT7* and *pGADT7* vectors. The yeast two-hybrid assay was performed using a Yeast Transformation System kit (Clontech, CA, USA) in accordance with the manufacturer’s protocol. The transformed AH109 yeast cells were grown on SD/-T-L and then incubated at 28°C for 3 days. The positive colonies were subsequently transferred into the selective and stringent SD/-T-L-H medium supplemented with 2 mM 3-AT medium.

For BiFC analysis, the coding region of GbSBT1 was cloned into pEarleyGate202 vector and PHB was cloned into pEarley-Gate201 vector. These vectors were transformed into the Agrobacterium strains EHA105. Equal volume suspensions of different Agrobacterium strains carrying *GbSBT1*-pEarleyGate202, PHB- pEarley-Gate201, p19 protein were mixed prior to infiltration. The re-suspended cells were infiltrated into leaves of tobacco plants as described previously. The fluorescence signals in infiltrated leaves were analyzed through confocal microscopy (Leica TCS SP5).

## Results

### Identification of the *GbSBT1* gene and phylogenetic analysis

Our previous study revealed more than 100 differentially expressed genes from the *Verticillium*-resistant cotton variety 7124 by using subtractive suppression hybridization [15]. Among these differentially expressed genes, the cDNA upregulated by *V*. *dahliae* attack was further studied in this work. The full-length cDNA was cloned by RT-PCR and was confirmed by DNA sequencing. This gene encoded a protein containing an evolutionary conserved structure that is highly similar to the subtilase proteins of other plants; thus, this gene was named *GbSBT1* and was deposited in GenBank (Accession NO: KT336228).

**Fig 1:**
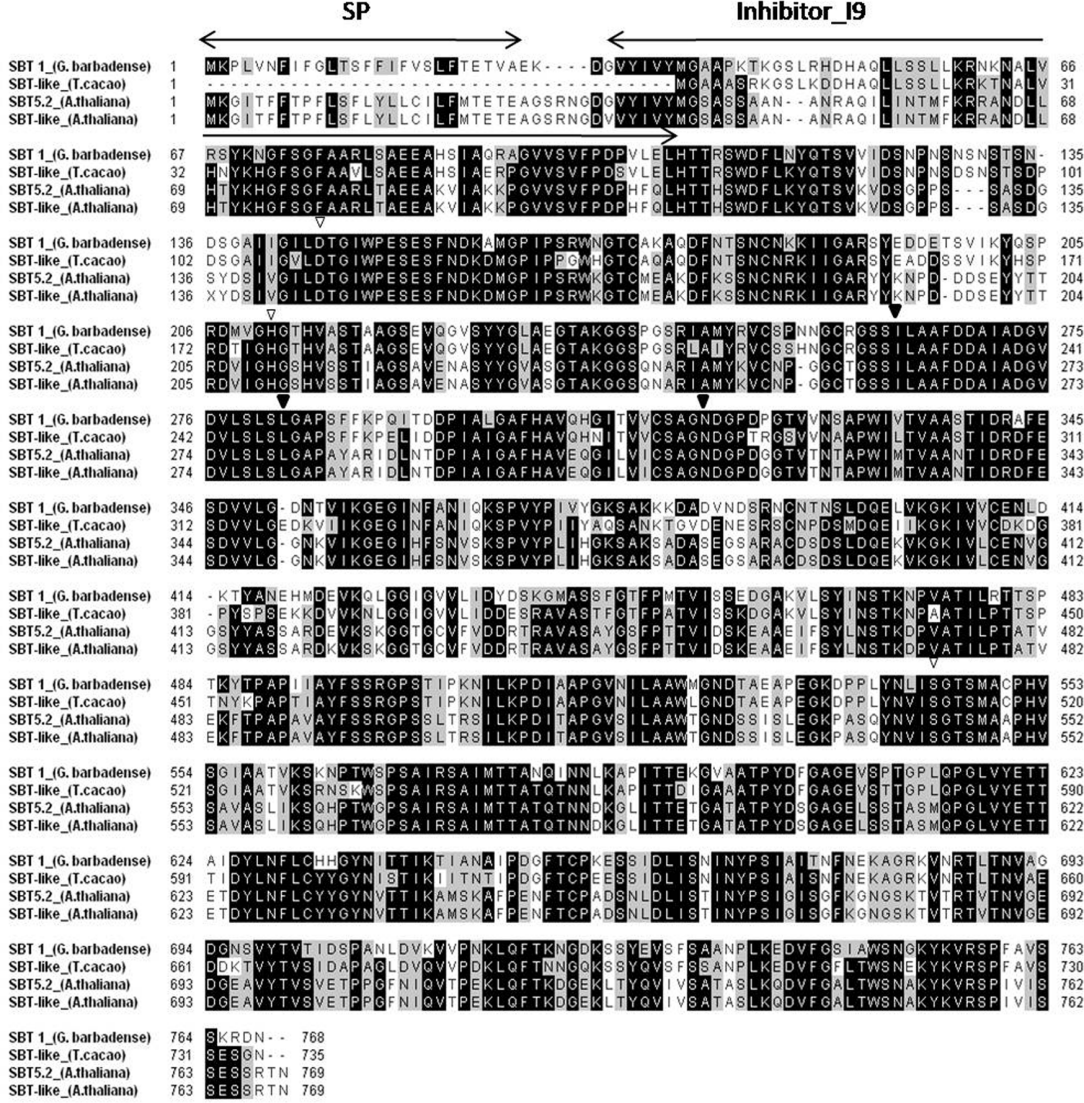
Multiple sequence alignment analysis of GbSBT1 and other plant subtilases. GbSBT1 (KT336228, *Gossypium barbadense*), subtilase-like (XP_007017870, *Theobroma cacao*), SBT5.2 (NP_564107, *Arabidopsis thaliana*), SBT-like (AAM65424, *A*. *thaliana*). SP: signal peptide; Inhibitor I9: serine protease inhibitor domain; black triangles indicate conserved active sites; blank triangles indicate catalytic triads of serine protease; and blank box A indicates PA/protease or protease-like domain interface.

**Fig 2:**
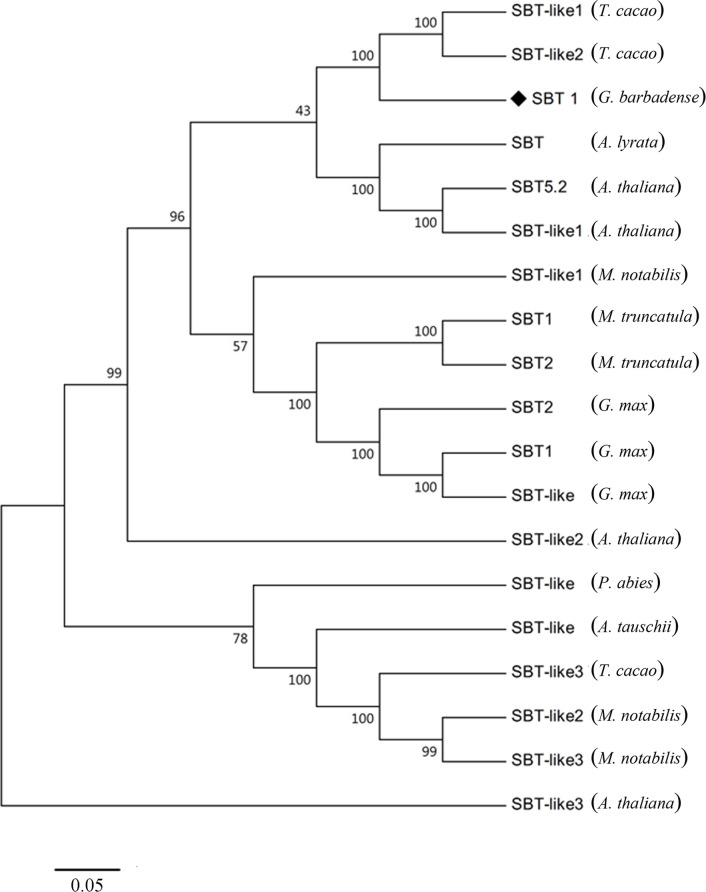
Phylogenetic analysis of GbSBT1 including the subtilases of different plant species. Nineteen of the complete amino acid sequences of subtilases were used to generate the neighbor–joining tree, and the numbers next to each node provide bootstrap values from 1000 replicates. The *GbSBT1* gene is marked by “◆.” GbSBT1 (KT336228, *Gossypium barbadense*), SBT-like *Picea abies* (BAA13135, *Picea sitchensis*), AtSBT5.2 (NP_564107, *Arabidopsis thaliana*), SBT-like1 *Theobroma cacao* (XP_007017870, *T*. *cacao*), SBT-like2 *T*. *cacao* (XP_007017871, *T*. *cacao*), SBT *Arabidopsis lyrata* (XP_002893091, *A*. *lyrata*), SBT-like1 *A*. *thaliana* (AAM65424, *A*. *thaliana*), SBT-like1 *Morus notabilis* (EXB60669, *M*. *notabilis*), SBT1 *Medicago truncatula* (XP_003603196, *M*. *truncatula*), SBT2 *M*. *truncatula* (XP_003603807, *M*. *truncatula*), SBT1 *Glycine max* (NP_001236511, *G*. *max*), SBT2 *G*. *max* (NP_001238252, *G*. *max*), SBT-like *G*. *max* (AAK53589, *G*. *max*), SBT-like2 *A*. *thaliana* (NP_564106, *A*. *thaliana*), SBT-like *Aegilops tauschii* (EMT32761, *A*. *tauschii*), SBT-like3 *T*. *cacao* (XP_007028364, *T*. *cacao*), SBT-like2 *M*. *notabilis* (EXB44295, *M*. *notabilis*), SBT-like3 *M*. *notabilis* (EXB44294, *M*. *notabilis*), SBT-like3 *A*. *thaliana* (NP_565309, *A*. *thaliana*).

The *GbSBT1* gene encodes a protein containing 769 aa, corresponding to a molecular mass of 81.78 kDa and a pI of 6.34. The GbSBT1 protein contains a putative signal peptide, an inhibitor domain, five conserved active sites (Ser-544, Asp-145, and His-223), and two catalytic triads of serine protease (Fig 1). A neighbor-joining phylogenetic tree of 19 subtilases indicated that that GbSBT1 is closely related to AtSBT5.2 (Fig 2). In *Arabidopsis*, AtSBT5.2 exhibits oxido-reductase activity and responds to an external biotic stimulus [10], indicating that the *GbSBT1* gene may function in biotic stress response.

### Expression pattern of *GbSBT1* gene following *V*. *dahliae* inoculation

**Fig 3:**
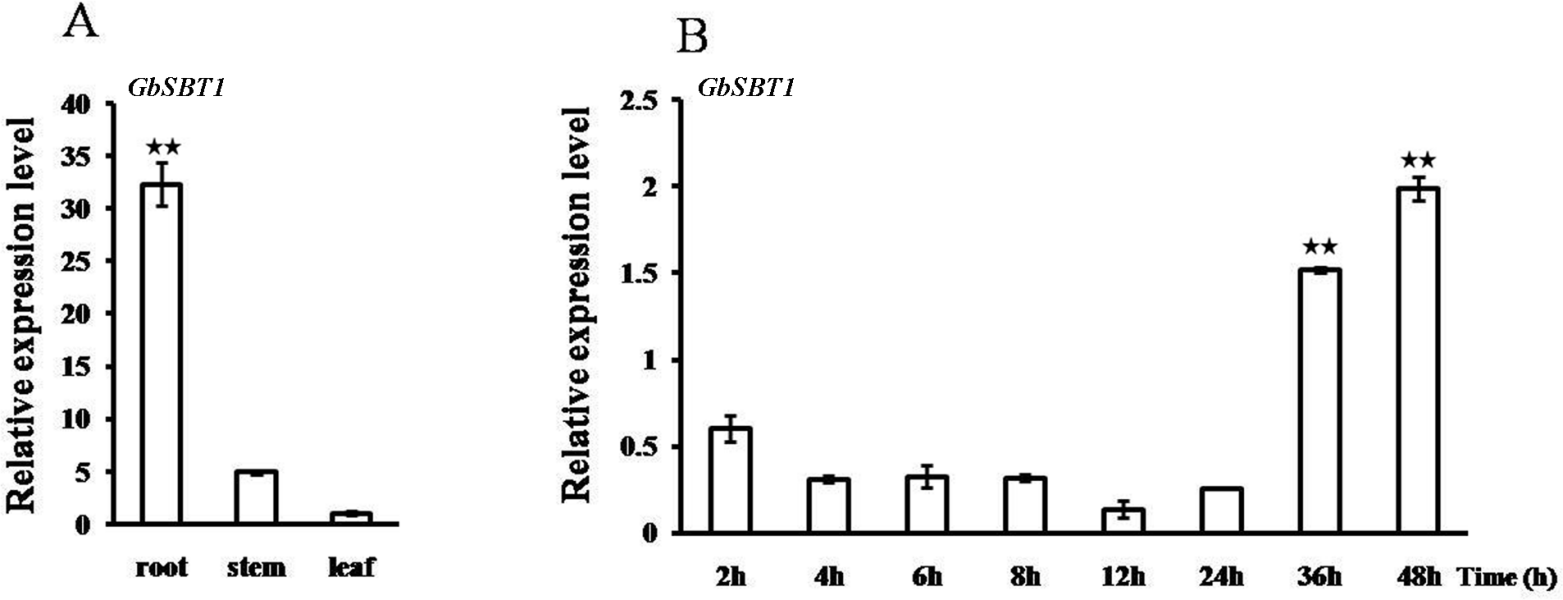
Induced *GbSBT1* expression following *V*. *dahliae* V991 infection. (A) Expression analysis of the *GbSBT1* gene in vegetative tissues (roots, stems, and leaves) via qPCR. The *Ubiquitin* gene is used as an internal control. At least three biological replicates were performed. (B) Expression analysis of *GbSBT1* in roots following *V*. *dahliae* strain V991 infection. The comparative CT method is adopted, and the expression is normalized. Each sample was repeated at least thrice. Error bars represent SE. Double asterisks represent a significant expression change of *GbSBT1* compared to uninfected condition (P < 0.01) in t-test.

qPCR was used to analyze the expression pattern of *GbSBT1* in various tissues of sea-island cotton. Fig 3A shows that the *GbSBT1* gene was ubiquitously expressed in all of the tested tissues. Among the tested tissues, *GbSBT1* expressed much higher in root where the *V*. *dahliae* enters through the roots more often than other organs.

In *Arabidopsis*, the induction of the *SBT3*.*3* gene was transient; that is, its expression peaked at 48 h post-inoculation (hpi) of the bacterial pathogen *Pseudomonas syringae* DC3000 and then abruptly decayed thereafter [11]. When cotton plants were inoculated with the V991 strain, *GbSBT1* expression in the roots decreased within 24 h, significantly increased thereafter, and then peaked at 48 hpi (Fig 3B). The rapid increase in *GbSBT1* expression suggests that *GbSBT1* is associated with cotton resistance to *Verticillium*.

### GbSBT1 protein is localized in the plasma membrane and is affected by jasmonic acid (JA) and ethylene

**Fig 4:**
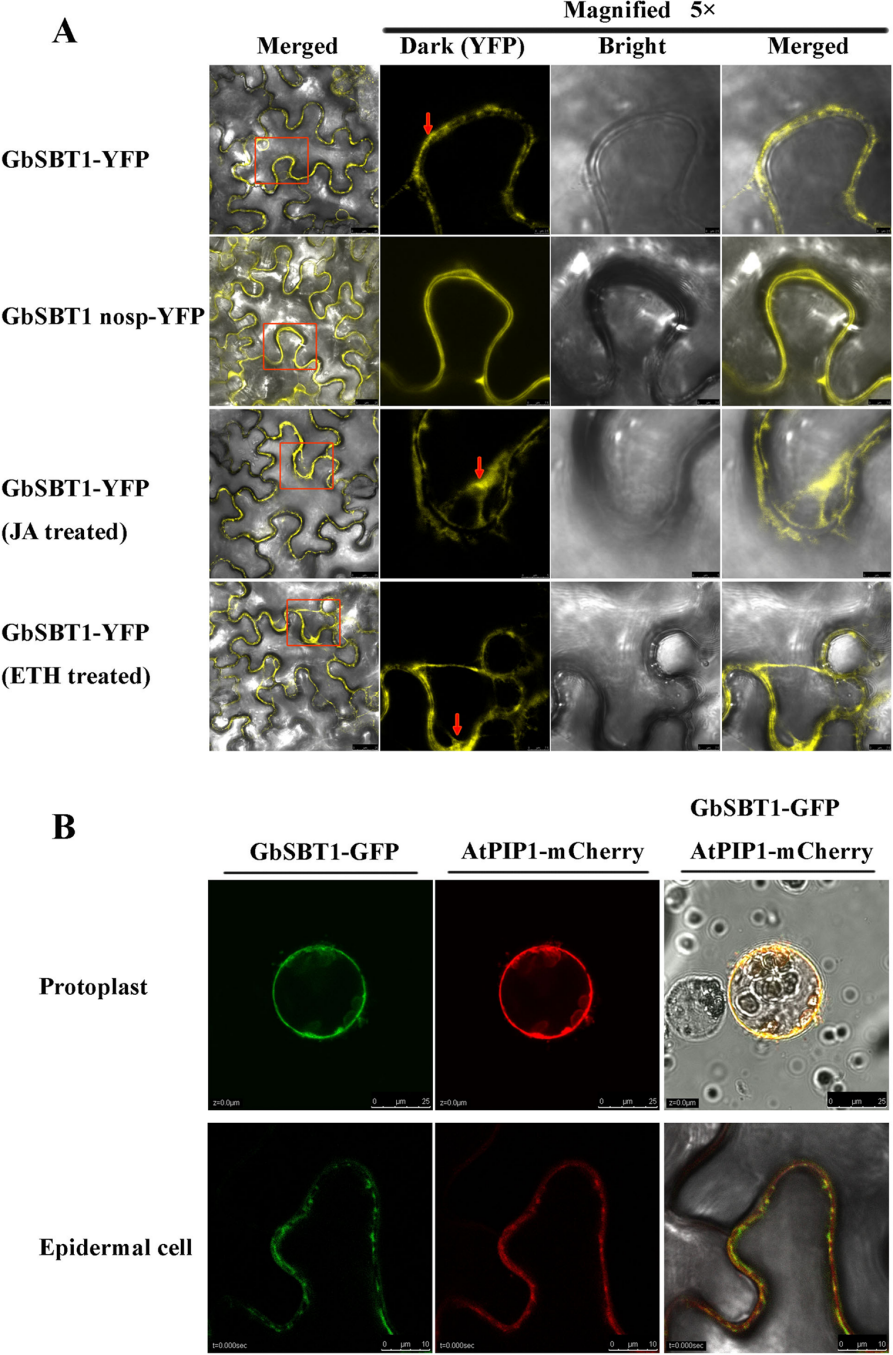
Extracellular localization of the GbSBT1 protein in tobacco leaves determined through confocal microscopy. (A) Localization of GbSBT1-YFP in tobacco leaves; localization of GbSBT1 (without signal peptide)-YFP in tobacco leaves; localization of GbSBT1-YFP in tobacco leaves under JA treatment; localization of GbSBT1-YFP in tobacco leaves under ethylene treatment. Arrows indicate the difference in protein localization between normal and phytohormone treatments; (B) GbSBT1 protein co-localized with plasma membrane integral protein PIP1 on the cell membrane using protoplast and tobacco lead epidermal cells as the protein expressing materials. Upper panels: protoplast; lower panels: tobacco leaf epidermal cells.

To analyze GbSBT1 subcellular localization, we expressed the ORF of the *GbSBT1* gene fused with *eYFP* in tobacco leaves. As shown in Fig 4A, GbSBT1-eYFP fluorescence signals were detected in the plasma membrane. When magnifying the accumulation area, GbSBT1-eYFP signals were unevenly distributed in the cell membrane (Fig 4A), which provides the conduits for the exchange of informational molecules that are central to cell growth and defense response in plants. When GbSBT1 without the signal peptide was expressed in tobacco leaves, GbSBT1 (no SP)-YFP signals were uniformly distributed in the plasma membrane. The co-localization signals with plasma membrane integral protein PIP1-mcherry further demonstrate that GbSBT1 mostly targets the cell membrane (Fig 4B). Overall, GbSBT1 is mainly localized in the cell membrane. GbSBT1 extracellular localization may be linked to the acceptance of external signals.

Verticillium wilt *Ve1*-mediated resistance is compromised in the *Arabidopsis* JA response mutant jar1-1 [22], and the JA signaling pathway can be activated in cotton by inoculation with *V*. *dahliae* [19,23], suggesting that cotton *Verticillium* resistance is related to JA signal transduction. To elucidate whether or not phytohormones affect GbSBT1 localization, we treated the cotton seedlings with methyl jasmonate and ethylene. Similar to the normal condition, GbSBT1 was unevenly localized in the cell membrane prior to the treatments with ethephon (1 mM) and JA (10 ppm). GbSBT1-YFP signal was induced and moved into cytoplasm after spraying these phytohormones (Fig 4A). Tracking the movement of the GbSBT1-YFP complex *in vivo* revealed that GbSBT1 particles rapidly moved along microfilaments in the cytoplasm (S1 Video). The movement of the GbSBT1 protein from the cell membrane into the cytoplasm suggests that GbSBT1 senses the combined signals of ethylene and JA treatments.

### Knockdown of *GbSBT1* gene expression results in lost resistance of cotton plants to *V*. *dahliae* infection

**Fig 5:**
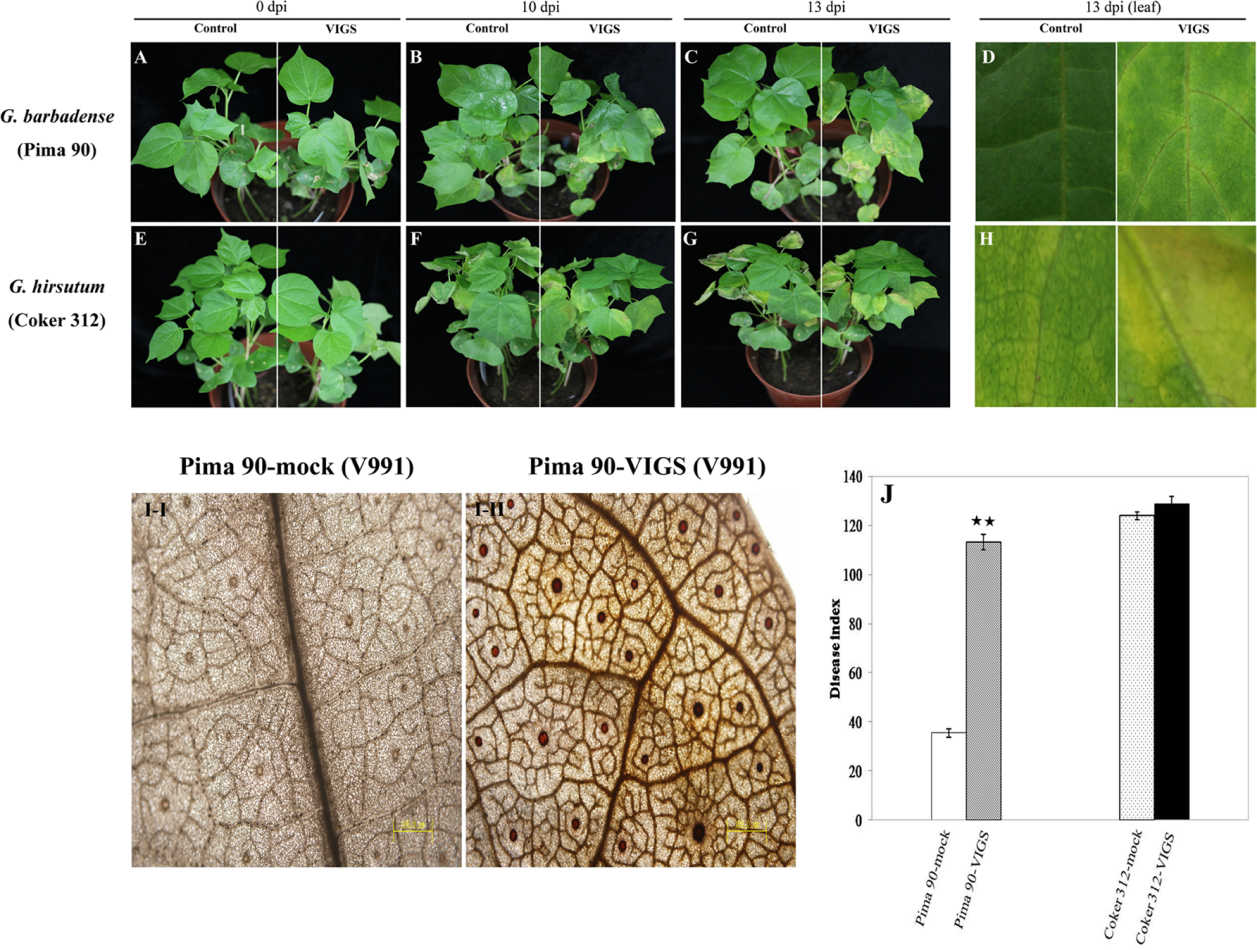
Reduced resistance of cotton to *Verticillium dahliae* after *GbSBT1* gene silencing. (A–C) Disease symptoms in *Gossypium barbadense* Pima-90 and the VIGS plants inoculated with *V*. *dahliae* strain V991; (D) Enlarged region of disease symptoms on leaf of Pima-90 inoculated with *V*. *dahliae* strain V991. Left panels: Pima-90; right panels: Pima-90 containing silenced *GbSBT1* gene; dpi: days post-inoculation. (E–G) Disease symptoms in *Gossypium hirsutum* Coker-312 and the VIGS plants inoculated with *V*. *dahliae* strain V991; (H) Enlarged region of disease symptoms on leaf of Coker-312 inoculated with *V*. *dahliae* strain V991. Left panels: Coker-312; right panels: Coker-312 containing silenced GhSBT1 gene; dpi: days post-inoculation. (I–I/II) DAB staining in the control and VIGS cotton leaves of *G*. *barbadense* after *V*. *dahliae* V991 inoculation. (J) Disease index measurement in Pima-90 and Coker-312 cotton plants after inoculation with V991. VIGS experiments were repeated at least three times with more than 10 cotton plants for each construct. Double asterisks represent significant difference between VIGS plants and wild-type plants (P < 0.01) in t-test.

We generated *GbSBT1* gene-silenced cotton plants through VIGS to study the function of the *GbSBT1* gene during *V*. *dahliae* infection. After growing in a greenhouse for 7 days, the *V*. *dahliae* resistance of the control (Pima-90) and VIGS plant lines was subsequently examined and quantified. qPCR results showed that *GbSBT1* was successfully repressed in VIGS plants. Results indicated that GbSBT1 expression in the VIGS plants was repressed significantly (about 76% lower than control) compared with control plants (S1 Fig). The *GbSBT1* gene-silenced and control plants were inoculated with *V*. *dahliae* strain V991. As shown in Fig 5A, *GbSBT1*-silenced plants grew as normal as the control plants before fungal inoculation. Ten days after inoculation, the silenced plants began to display disease symptoms in the form of margin-wilted leaves; by contrast, the control cotton plants grew normally (Fig 5B). Thirteen days after inoculation, the *GbSBT1*-silenced plants were severely affected and the majority of leaves turned yellow and tended to wilt (Fig 5C, right panel). Moreover, the rate of diseased plants and the disease index were significantly higher in the *GbSBT1*-silenced plants than in the control plants (Fig 5J). This result suggests that *GbSBT1* knockdown in cotton plants increases plant susceptibility to *V*. *dahliae* infection.

The *subtilase 1* gene in Coker-312 was further silenced and the disease symptom was investigated to validate the effect of *subtilase 1* gene expression on *V*. *dahliae* resistance in different genotypes (resistant: Pima-90; sensitive: Coker-312). Ten days after inoculation, the *GhSBT1*-silenced and control plants were both severely affected, and the bottom leaves began to defoliate (right panel, Fig 5E). As the infection progressed, the control and silenced plants still showed no obvious difference in symptoms. The disease index showed no significant difference between the control and VIGS plants (Fig 5J). These results demonstrated that *subtilase1* suppression in the sensitive genotype failed to facilitate *V*. *dahliae* infection and that the *GbSBT1* gene is involved in R-gene resistance.

Reactive oxygen accumulation is related to hypersensitive cell death, which could lead to the spontaneous formation of lesions in plants. In addition, H_2_O_2_ accumulation could impede *V*. *dahliae* infection in cotton [5]. The changes in H_2_O_2_ levels in the control and VIGS cotton plants were observed through DAB staining to investigate whether or not high levels of H_2_O_2_ are produced during *V*. *dahliae* infection. As shown in Fig 5I, control Pima-90 plants have no DAB staining. Conversely, the VIGS plants showed obvious DAB staining in the leaf vessels, indicating that *V*. *dahliae* pathogens ingress into cotton plants and induce H_2_O_2_ accumulation.

### Ectopically expressed *GbSBT1* gene in *Arabidopsis* confers disease resistance to *F*. *oxysporum* and *V*. *dahliae*

**Fig 6:**
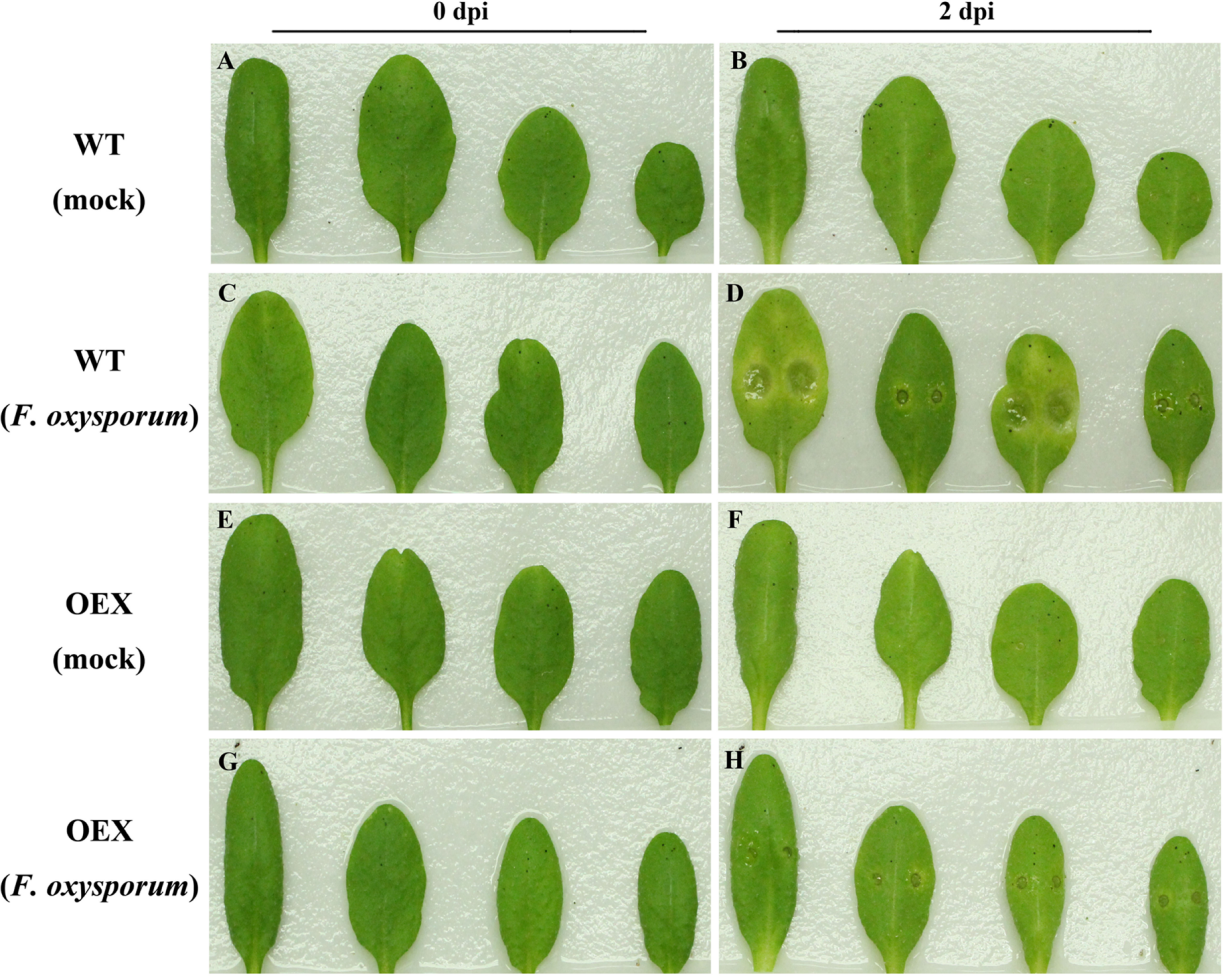
Enhanced disease resistance of *Arabidopsis* against *Fusarium oxysporum* after *GbSBT1* overexpression. Wild-type (WT): *Arabidopsis* Columbia type Col-0; OEX: transgenic *GbSBT1* lines. Left panels: un-inoculated plants; right panels: plants 2 days after *F*. *oxysporum* inoculation. (A–B) WT plants 0 and 2 days after ddH_2_O inoculation; (C–D) WT plants 0 and 2 days after *F*. *oxysporum* inoculation; (E–F) transgenic *GbSBT1* plants 0 and 2 days after inoculation with ddH_2_O containing 0.2% Tween-20; (G–H) transgenic *GbSBT1* plants 0 and 2 days after *F*. *oxysporum* inoculation. At least three biological replicates were performed in the *F*. *oxysporum* resistance analysis.

**Fig 7:**
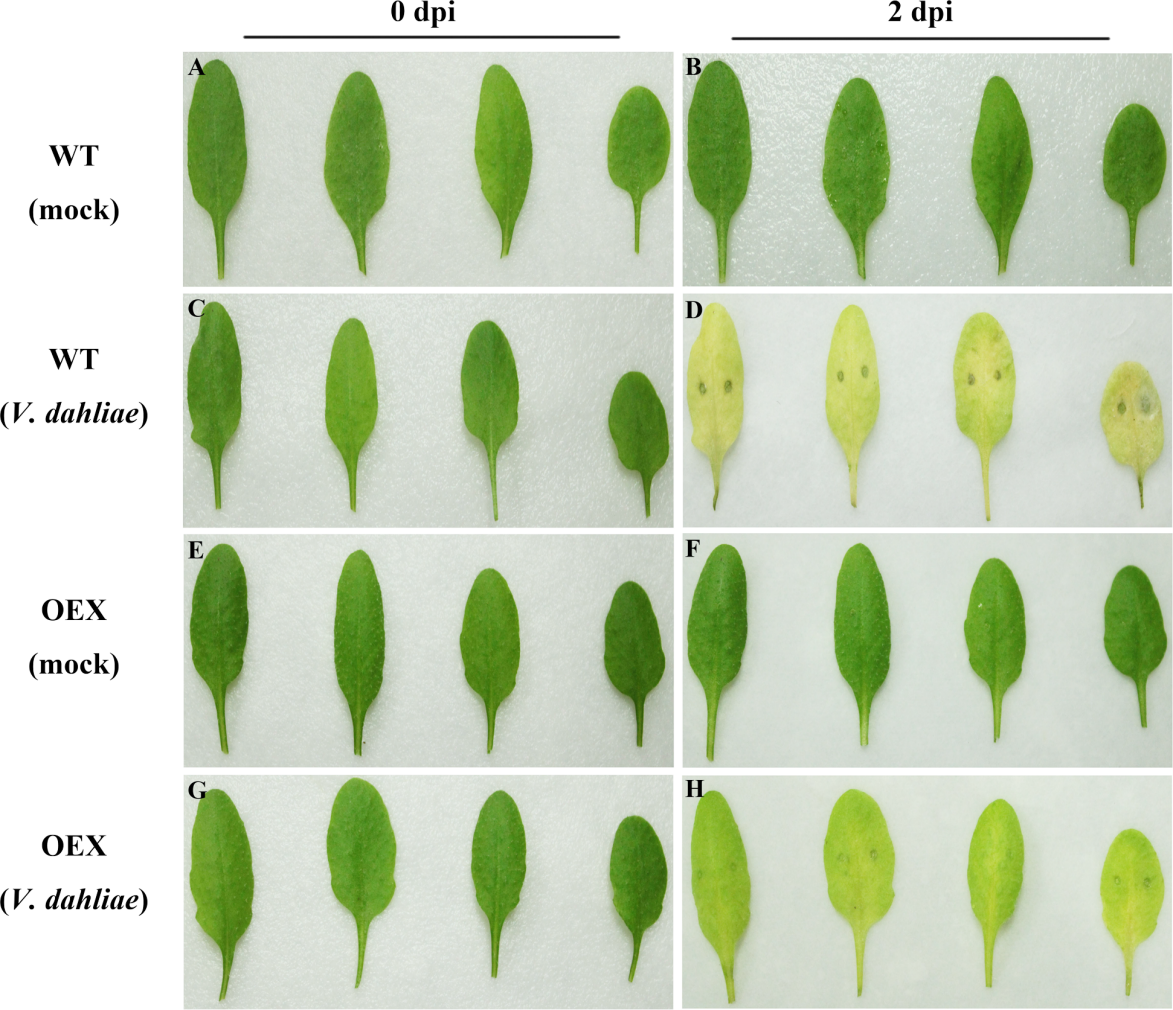
Enhanced disease resistance of *Arabidopsis* against *V*. *dahliae* after *GbSBT1* overexpression. Wild-type (WT): *Arabidopsis* Columbia type Col-0; OEX: transgenic *GbSBT1* lines. Left panels: un-inoculated plants; right panels: plants 2 days after *V*. *dahliae* inoculation. (A–B) WT plants 0 and 2 days after ddH_2_O inoculation; (C–D) WT plants 0 and 2 days after *V*. *dahliae* inoculation; (E–F) transgenic *GbSBT1* plants 0 and 2 days after inoculation with ddH_2_O containing 0.2% Tween-20; (G–H) transgenic *GbSBT1* plants 0 and 2 days after *V*. *dahliae* inoculation. At least three biological replicates were performed in the *V*. *dahliae* resistance analysis.

**Fig 8:**
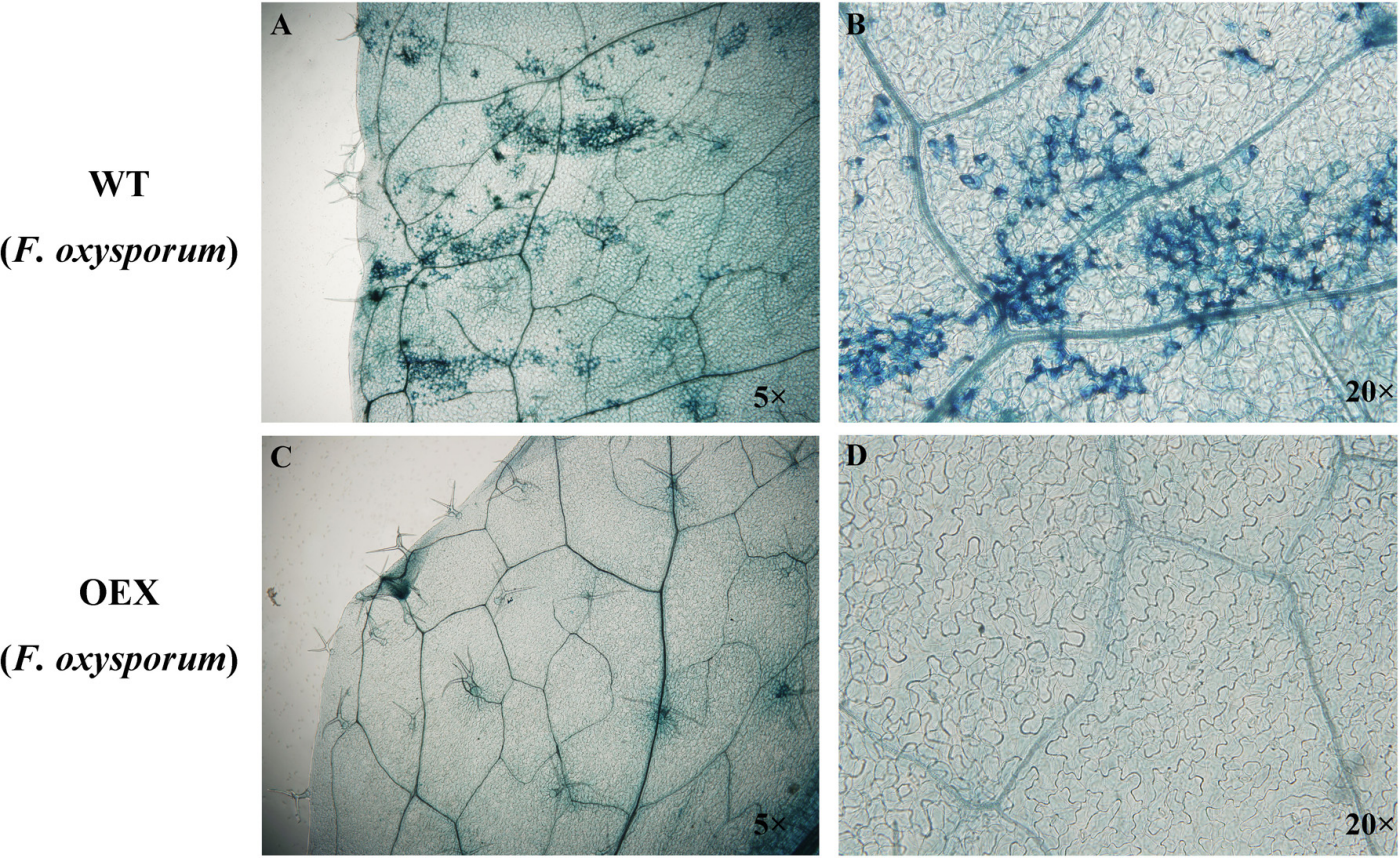
Trypan blue staining of *Fusarium oxysporum*-inoculated leaves. (A) Wild-type (WT) plants inoculated with *F*. *oxysporum*; (B) 20×magnification of the infected area in WT; (C) *GbSBT1* overexpression in *F*. *oxysporum-*inoculated *Arabidopsis* leaves; (D) 20×magnification of the infected area in *GbSBT1*-overexpressing *Arabidopsis*. The trypan blue staining experiments were repeated at least three times.

To better understand the molecular mechanisms of *GbSBT1* in the immune response, we generated and self-crossed transgenic *GbSBT1 Arabidopsis* plants. After three rounds of self-crossing, two homologous lines showing different expression levels of the *GbSBT1* gene were randomly selected and subjected to *F*. *oxysporum* and *V*. *dahliae* challenge. qPCR analysis of *GbSBT1* expression levels in transgenic *Arabidopsis* lines OEX1 and OEX2 were carried out (S2 Fig). Obvious differences in disease symptoms between the WT and transgenic plants were observed at 2 days post-inoculation (dpi). The necrotic lesions were larger in the WT leaves than in the transgenic leaves (Fig 6). The disease spot diameter was also significantly smaller in the transgenic plants (0.36 ± 0.31 mm) than in the control plants (2.52 ± 0.66 mm). Similarly, necrotic lesions of *V*. *dahliae* on *Arabidopsis* leaves were smaller in the transgenic plants than in the WT plants (Fig 7). This result suggests that *GbSBT1* overexpression inhibits the spread of necrotic lesions in transgenic leaves. Moreover, the control plants showed more pathogen aggregates near their leaf vessels, whereas the transgenic plants showed a negative result for trypan blue staining (Fig 8). As the infection progressed, the control plants wilted, whereas the transgenic *GbSBT1* plants grew normally. These results demonstrate that the ectopic expression of *GbSBT1* confers the increased tolerance of *Arabidopsis* against *F*. *oxysporum* infection.

### Overexpression of *GbSBT1* gene in *Arabidopsis* enhances the expression of defense-related genes

**Fig 9:**
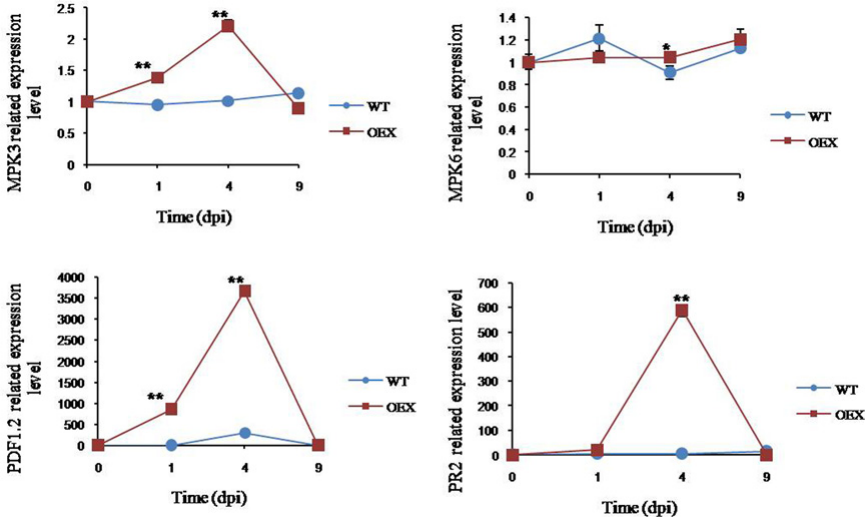
qPCR analysis of gene expression in WT and transgenic *GbSBT1* lines after *F*. *oxysporum* infection. Gene expression levels were normalized to the constitutive *ACT2* gene expression. Data represent the mean of gene expression levels with SD at different infection stages; n = 3 replicates. dpi: days post-inoculation. Error bars represent the standard deviation for three biological experiments, and three technical replicates were analyzed. Statistical significance was determined using two-tailed unpaired Student’s t-tests, and P values <0.05 were considered statistically significant (** for P values <0.01).

Given their consistent resistance effects on *GbSBT1*-overexpressing *Arabidopsis* plants and VIGS cotton plants, the transcripts of the genes involved in JA and immune transduction were analyzed in *Arabidopsis*. The expression levels of *PR-2* and *PDF1*.*2* were upregulated in the overexpressing lines following *F*. *oxysporum* inoculation (Fig 9). *PR-2* was induced, peaked at 4 dpi, and then abruptly decreased. The highest level of *PR-2* expression was nearly 10 times that of the WT plants at 4 dpi. *PDF1*.*2* induction was peaked at 4 dpi; the highest expression of this gene was approximately 98.8 times as that of the control plants. The increased expression of *PR*s in *GbSBT1* OEX lines contributed to the resistant phenotype.

The activation of mitogen-activated protein kinases (MAPKs) is linked to innate immune responses [24,25]. The expression levels of *MPK6* and *MPK3* were assessed in the WT plants and *GbSBT1* OEX lines to demonstrate whether or not the enhanced resistance phenotype is mediated by *GbSBT1* expression (Fig 9). After *F*. *oxysporum* inoculation, the expression levels of *MPK6* and *MPK3* were higher in the *GbSBT1* OEX lines than in the WT plants. *MAPK* activation in the *GbSBT1* OEX lines indicates that *GbSBT1* is a positive regulator of induced resistance response.

### GbSBT1 interacts with PHB protein secreted by *V*. *dahliae* strain V991

The GbSBT1-interacting proteins were identified using GFP monoclonal antibody by extracting the total proteins of the *V*. *dahliae*-inoculated cotton leaves to characterize whether or not GbSBT1 senses the infection signals from pathogens, the total proteins of cotton leaves inoculated with *V*. *dahliae* were extracted to identify the GbSBT1 interacting proteins using GFP monoclonal antibody. The pull-down results indicated that 128 proteins were characterized after NLM analysis including 15 secreted proteins and 113 unsecreted proteins (S2 Table). Gene ontology showed that these secreted proteins were assigned to biological processes, including metabolic process, pigmentation, and response to stimulus. After searching the functions of the homologes online, 15 secreted proteins containing a 15–30 aa signal peptide were found to be involved in protein translocation across organelles or cell membrane (Table 1). Gene enrichment analysis also revealed that most of the secreted proteins participated in signal transduction and protein transport. The Het-C protein (gi|697081603), similar to the sugar ester transfer protein, drastically impairs ascospore production [26]. In summary, most of the secreted proteins are involved in cell wall biogenesis and hyphal growth during pathogenic ingression into host plants.

**Table 1:** GbST1-interacting proteins secreted from *V*. *dahliae* V991.

Protein accession	Protein putative identity	Signal peptide	Peptide	Score
gi|697086726	peptidyl-tRNA hydrolase	MSDTLHLILTTSAVTFLSGFALGVFAI	13	378
gi|697069413	prohibitin	MAAALNFISKAAVPAFFGASLLSTAI	3	95
gi|697065929	acetyl-CoA acetyltransferase	MANLPSVYIVSAARTPVGSFLGSLSSLSAV	3	75
gi|697067287	oligosaccharyltransferase alpha subunit	MRAFAIATGLLSLVSAAVASSD	4	60
gi|697067481	carbamoyl-phosphate synthase arginine-specific large chain	MPSAMACSLAGRAPAVLRHGRRMALPVRSFTSLTAASKSFTPAQSQ	3	59
gi|697088233	zinc transporter SLC39A9	MGGLFLLLVLCAVMAVASFLAG	4	59
gi|697067381	uridylate kinase	MHATPRTLWRSLPTACTASRIASST	4	54
gi|697086240	phosphoribosylaminoimidazole-succinocarboxamide synthase	MSSSAPLTTLSLPSLERIASGK	2	44
gi|697066901	disulfide-isomerase erp38	MVLLKSFVLGALAATAAAK	3	36
gi|697068043	benzodiazepine receptor family protein	MTTFIPALTLPRQVFDHPATSILLPIALGTAVGYTSSR	3	29
gi|697089965	CTP synthase	MKYLLVSGGVISGPLRRAFCS	2	28
gi|697081603	Het-C protein	MASFHFGKGSWFLVFCIVLVLLPGRAAAF	2	25
gi|697068103	helicase SWR1	MAGLAAGFSSSPPGRGF	2	20
gi|697071463	short-chain dehydrogenase/reductase SDR	MAAHRSATRALARAFTARTAAA	2	18
gi|697077881	hypothetical protein VDAG_09776	MSSNTLDNTALLATGICMVILTSAVFGF	2	18

The proteins listed here were all detected at least thrice. The signal peptides were predicted by SignalP 4.1 online. “Peptide” indicates the number of detected peptide in the putative protein. Putative proteins were removed if number of detected peptide less than two (Peptide<2).The score indicates the total number of detection in the pellet pulled down by the antibody after digestion.

**Fig 10:**
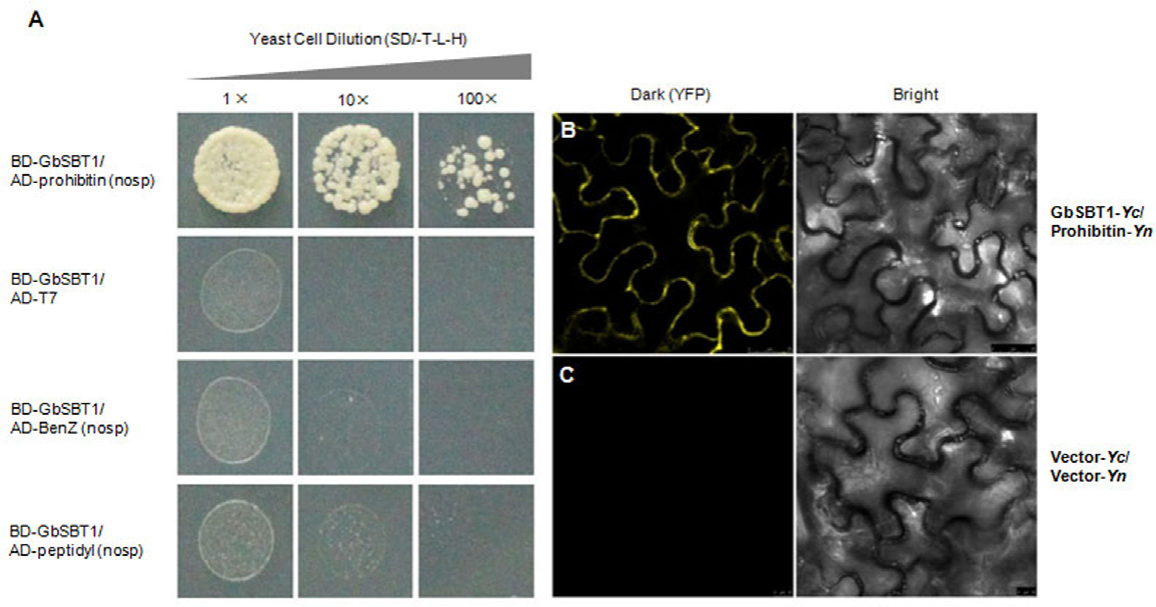
Interaction between GbSBT1 and prohibitin secreted by *Verticillium dahliae*. (A) The yeast cells co-expressing GbSBT1 and benzodiazepine receptor family protein or peptidyl-tRNA hydrolase could not grow on the selective medium SD/-T-L-H, whereas the yeast cells co-transformed with GbSBT1 and prohibitin could grow on the selective medium SD/-T-L-H; (B-C) BiFC of epidermal cells co-expressing split YFP fusions of *GbSBT1* and prohibitin or empty vector controls. Combinations of N- and C-terminal YFP fragments (Yn and Yc, respectively) were infiltrated as vector controls or fused to the N terminus of *GbSBT1* and prohibitin as follows: B: *GbSBT1*-Yc and prohibitin -Yn, Scale bar: 25 μm; C: vector-Yc and vector-Yn, Scale bar: 10 μm. The interaction and co-localization were observed at the plasma membrane. Right is the corresponding bright-field

To further confirm which protein directly interacts with GbSBT1 during *V*. *dahliae* ingression into cotton, three proteins (based on the number of detected peptides in pull-down assay) were tested their interactions with GbSBT1 in yeast-two-hybridization. As shown in Fig 10A, GbSBT1 did not interact with the benzodiazepine receptor family protein and peptidyl-tRNA hydrolase, whereas the yeast cells co-transformed with GbSBT1 and PHB grew on the selective medium SD/-T-L-H. This result indicates that GbSBT1 can directly bind to PHB.

Bimolecular fluorescence complementation (BiFC) analysis was used to confirm the interaction between GbSBT1 and PHB in vivo. Fluorescence formed through formation of a fluorescent complex comprising of two fragments of YFP could be observed in the plasma membrane of tobacco leaf cells, indicating that PHB and GbSBT1 actually interacted in plants and as predicted (Fig 10B and 10C).

## Discussion

*V*. *dahliae* secretes various effectors for its invasion and colonization in cotton. For instance, *V*. *dahliae* secretes pectinase or xylanase to degrade the cell wall of the elements of the vascular system and disrupt water transport in cotton [7]. The isochorismatase VdIsc1 is also secreted by *V*. *dahliae* to suppress the salicylate-mediated innate immunity of host cells [4]. Although the effector candidates have been identified in the *V*. *dahliae* genome and the functions of several effectors have been recently characterized, little is known about how the cotton plant interferes with the infection and colonization of the filamentous pathogen *V*. *dahliae*. In this study, we analysis the function of *GbSBT1* and provide the evidences that GbSBT1 is involved in *Verticillium*-induced resistance in cotton.

Host proteases are important defense components against pathogens [27]. Host plants use proteases to monitor or facilitate effector-mediated degradation and subsequently activate effector-triggered immunity [28,29,30]. The extracellular subtilase P69C specifically processes an extracellular leucine-rich repeat LRP protein in tomato to initiate various signaling processes [31]. In *Arabidopsis*, *SBT3*.*3* expression initiates a durable auto-induction mechanism that activates the salicylate-dependent expression of defense genes for amplified immune response [11]. Whether or not the GbSBT1 protein activates immune response remains unknown. Similar to the reported subtilases, GbSBT1 is localized in the cell membrane and in the extracellular apoplast, where *V*. *dahliae* ingression into cotton plant occurs and which serves as the recognition site between cotton plant and *V*. *dahliae*. In contrast to the *SBT3*.*3* gene in *Arabidopsis*, the *GbSBT1* gene is activated by JA and ethylene treatments instead of SA stimulus, demonstrating that JA signaling is required for the resistance of cotton plant against *V*. *dahliae* [23]. Moreover, the GbSBT1 protein moves from the cell membrane into the cytoplasm after *V*. *dahliae* attack and JA treatment, indicating that *GbSBT1* is related to JA-induced resistance against *V*. *dahliae* [23].

The JA-responsive gene *PDF1*.*2* encodes a protein that exhibits antimicrobial activity, which is dependent on COI1 and EIN2, and is correlated with resistance to necrotrophic pathogens [32,33]. Increased *PDF1*.*2* expression indicated that *GbSBT1* overexpression in *Arabidopsis* can induce the JA signaling pathway when responding to *F*. *oxysporum* attack. *GbSBT1* overexpression in *Arabidopsis* also enhanced resistance against *F*. *oxysporum* and upregulated the expression of the *PR1* and *PR2* genes after fungal inoculation. Correspondingly, *GbSBT1* knockdown in Pima-90 resulted in lost resistance to *V*. *dahliae* and reduced expression of the *PR* genes (S3 Fig). The changes in the expression of *PR* genes in both *Arabidopsis* and cotton plants indicate that *GbSBT1* gene expression activates induced resistance. Furthermore, the *MPK6* and *MPK3* genes that are associated with innate immune response were activated by *GbSBT1* gene expression in *Arabidopsis*, demonstrating that *GbSBT1* plays important roles in innate immune responses. Considering that the SBT1 protein has no amino acid difference between from Coker-312 (sensitive to *V*. *dahliae*) and Pima-90 (resistant to *V*. *dahliae*), so we silenced the *SBT1* gene in two genetic backgrounds and investigated their disease symptoms. The lost resistance to *V*. *dahliae* in Pima-90 and the absence of severe susceptibility of Coker-312 shows that the *GbSBT1* gene is involved in R-gene resistance.

In tomato, cysteine protease is required for *Cf2*-dependent disease resistance and auto-necrosis suppression. During incompatible interaction, Avr2 physically interacts with and inhibits the extracellular Cys-protease Rcr3 in resistant tomato varieties [34]. The localization of GbSBT1 protein made it possible for its recognition with protein secreted by *V*. *dahliae*. Therefore, we employed the pull-down assay to identify GbSBT1-interacting proteins and thus analyze further the role of GbSBT1 in *Verticillium* resistance. A total of 15 secreted proteins in *V991* pathogen were identified as candidates. Searching for the functions of the homologs of these proteins revealed that the secreted proteins are likely related to the pathogenicity of *V*. *dahliae* during the invasion of host plants. The first type of secreted proteins, such as Het-C protein (gi|697081603), is required for hyphal and spore growth, and deletion of these genes results in the loss of cell wall integrity. The second type of secreted proteins facilitates pathogen evasion from host surveillance. All of the pull-down proteins of *V*. *dahliae* are involved in pathogenicity, indicating that GbSBT1 may effectively recognize the effectors of *V*. *dahliae*.

To confirm further which specific effector interacts with GbSBT1, we determined the interaction of three proteins in the yeast two-hybrid system and confirmed that PHB is the GbSBT1-interactor. PHB, which is localized in the cell membrane, is a highly conserved protein exhibiting a common structure and a domain that is similar to that of other organisms. In human, the PHB1 of the host binds to RtxA1-D2, and downregulation of *PHB1* genes by small interfering RNAs reduces RtxA1-D2 cytotoxicity against HeLa cells. PHB1 is directly involved in RtxA1 cytotoxicity when *Vibrio cholerae* infects HeLa cells [35]. Furthermore, the Vi capsular polysaccharide of *Salmonella typhi* targets the PHB family in intestinal epithelial cells and suppresses early inflammatory responses [36]. These findings reveal that PHB modulates early inflammatory responses during infection. In plants, PHB3 affects NO accumulation and NO-mediated processes [37]. Moreover, a *PHB3* mutant exhibits an extreme constitutive ethylene response in etiolated seedlings and reduces ethylene-inducible genes, such as *AtEBP* and *PDF1*.*2*, suggesting that AtPHB3 plays dual roles in ethylene signaling [38]. These findings indicate that PHB is associated with inflammatory response in human, and ethylene or NO mediated cellular responses in plants. In this study, we found that a PHB interacts with GbSBT1 by using pull-down and yeast two-hybrid assays. Based on the function of GbSBT1 during *Verticillium* ingression, we hypothesized that the PHB from *V*. *dahliae* interacts with GbSBT1 and then activates the transcription of genes related to *Verticillium* resistance signaling in cotton. However, the mechanism underlying GbSBT1-PHB interaction and the roles of PHB in *Verticillium* resistance warrant further investigation.

## Supporting Information

S1 Fig qPCR analysis of *GbSBT1* expression in Mock and VIGS plants. VIGS experiments were repeated at least three times with more than 10 cotton plants for each construct. Double asterisks represent significant difference between VIGS plants and wild-type plants (P < 0.01) in t-test.
(PDF)

S2 Fig qPCR analysis of *GbSBT1* expression levels in transgenic *Arabidopsis* lines OEX1 and OEX2. The comparative CT method is adopted, and the expression is normalized. Each sample was repeated at least thrice. Error bars represent SE. Double asterisks represent a significant expression change of GbSBT1 compared to uninfected condition (P < 0.01) in t-test.
(PDF)

S3 Fig qPCR analyses of *PR1* and *PR2* expression in *Gossypium babardense* (Pima-90) and its VIGS plants 10 days after *V*. *dahliae* strain V991 inoculation. Total RNA was isolated from leaves of the control and inoculated VIGS cotton plants. Changes in the expression of defense-related genes, including *PR1* and *PR2*, were analyzed via qRT-PCR. The ubiquitin7 gene (DQ116441) of cotton plant served as an internal control. The primers (*GbPR1* and *GbPR2*) used in qRT-PCR analysis are listed in S1 Table. Gene expression levels were normalized to the constitutive *Ubiquitin* gene expression. The expression levels are the means with SD; n = 3 replicates. dpi: days post-inoculation. Double asterisks represent a very significant difference between VIGS plants and wild-type plants (P < 0.01) in t-test.
(PDF)

S1 Table The sequence and primers used in this study. (DOCX)

S2 Table The total of proteins pulled down by GbSBT1. (DOCX)

S1 Video The movement of GbSBT1 particles from the cell membrane to the cytoplasm. (MP4)
