# Supplementary material for: Characterization of a Novel Cotton Subtilase Gene GbSBT1 in Response to Extracellular Stimulations and Its Role in Verticillium Resistance
Source: PLoS One. 2016 Apr 18;11(4):e0153988. doi: 10.1371/journal.pone.0153988 (PMC4835097; doi:10.1371/journal.pone.0153988)
Supplement: S1 Fig — VIGS experiments were repeated at least three times with more than 10 cotton plants for each construct. Double asterisks represent significant difference between VIGS plants and wild-type plants (P < 0.01) in t-test. (PDF) [file pone.0153988.s001.PDF]

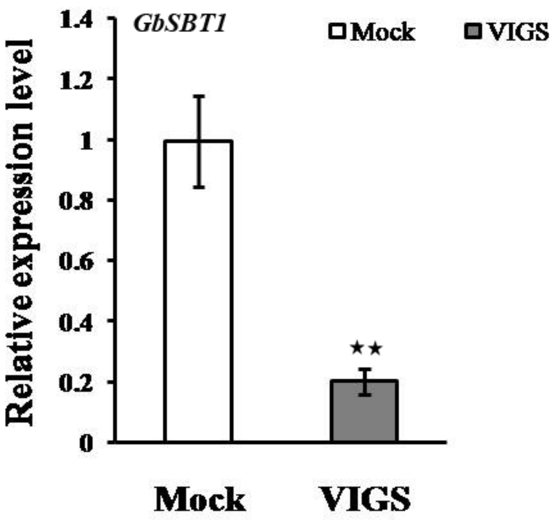

Supplementary Figure 1. qRT-PCR analysis of *GbSBT1* expression in Mock and VIGS plants. VIGS experiments were repeated at least three times with more than 10 cotton plants for each construct. Double asterisks represent significant difference between VIGS plants and wild-type plants ($P < 0.01$) in t-test.
