# Supplementary material for: Characterization of a Novel Cotton Subtilase Gene GbSBT1 in Response to Extracellular Stimulations and Its Role in Verticillium Resistance
Source: PLoS One. 2016 Apr 18;11(4):e0153988. doi: 10.1371/journal.pone.0153988 (PMC4835097; doi:10.1371/journal.pone.0153988)
Supplement: S2 Fig — The comparative CT method is adopted, and the expression is normalized. Each sample was repeated at least thrice. Error bars represent SE. Double asterisks represent a significant expression change of GbSBT1 compared to uninfected condition (P < 0.01) in t-test. (PDF) [file pone.0153988.s002.PDF]

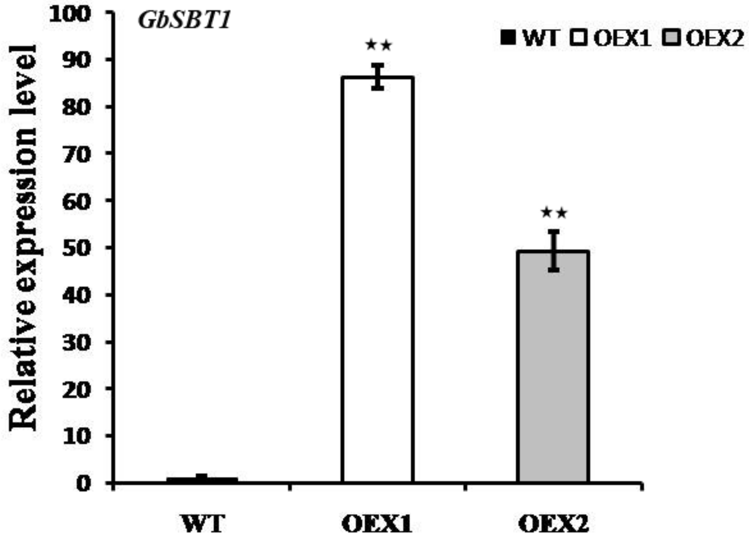

Supplementary Figure 2. qPCR analysis of *GbSBT1* expression levels in transgenic Arabidopsis lines OEX1 and OEX2. The comparative CT method is adopted, and the expression is normalized. Each sample was repeated at least thrice. Error bars represent SE. Double asterisks represent a significant expression change of GbSBT1 compared to uninfected condition ($P < 0.01$) in t-test.
