# Supplementary material for: Characterization of a Novel Cotton Subtilase Gene GbSBT1 in Response to Extracellular Stimulations and Its Role in Verticillium Resistance
Source: PLoS One. 2016 Apr 18;11(4):e0153988. doi: 10.1371/journal.pone.0153988 (PMC4835097; doi:10.1371/journal.pone.0153988)
Supplement: S3 Fig — Total RNA was isolated from leaves of the control and inoculated VIGS cotton plants. Changes in the expression of defense-related genes, including PR1 and PR2, were analyzed via qRT-PCR. The ubiquitin7 gene (DQ116441) of cotton plant served as an internal control. The primers (GbPR1 and GbPR2) used in qRT-PCR analysis are listed in S1 Table. Gene expression levels were normalized to the constitutive Ubiquitin gene expression. The expression levels are the means with SD; n = 3 replicates. dpi: days post-inoculation. Double asterisks represent a very significant difference between VIGS plants and wild-type plants (P < 0.01) in t-test. (PDF) [file pone.0153988.s003.pdf]

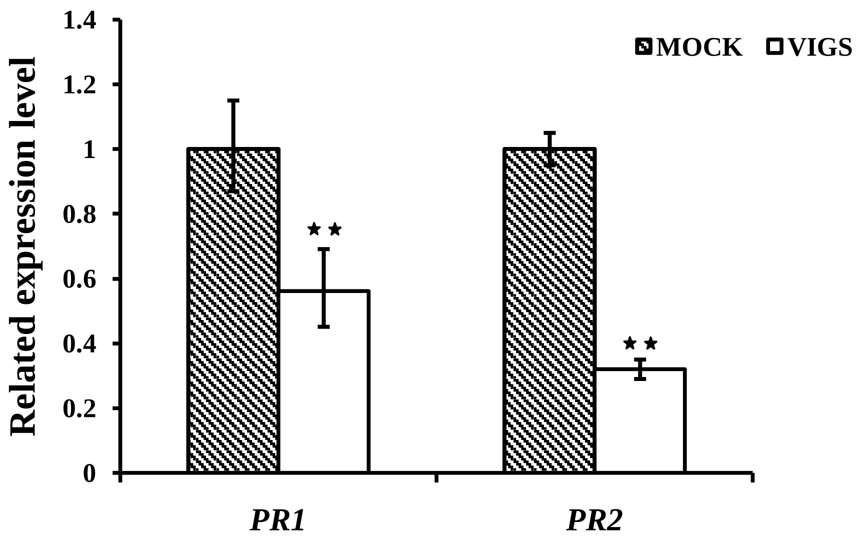

Supplementary Figure 3. qPCR analyses of *PR1* and *PR2* expression in *Gossypium babardense* (Pima-90) and its VIGS plants 10 days after *V. dahliae* strain V991 inoculation. Gene expression levels were normalized to the constitutive *Ubiquitin* gene expression. The expression levels are the means with SD; n = 3 replicates. dpi: days post-inoculation. Double asterisks represent a very significant difference between VIGS plants and wild-type plants ($P < 0.01$) in t-test.
