# Supplementary material for: Characterization of a Novel Cotton Subtilase Gene GbSBT1 in Response to Extracellular Stimulations and Its Role in Verticillium Resistance
Source: PLoS One. 2016 Apr 18;11(4):e0153988. doi: 10.1371/journal.pone.0153988 (PMC4835097; doi:10.1371/journal.pone.0153988)
Supplement: S1 Table — (DOCX) [file pone.0153988.s004.docx]

**Table S1. The sequence and primers used in this study.**

| **Assay** | **Gene** | | **Primer direction** | | **Primer sequence 5’-3’** |
| --- | --- | --- | --- | --- | --- |
| subcellular localization | *GbSBT1* | Forward | | GGGGACAAGTTTGTACAAAAAAGCAGGCTTCATGAAGCCCCTCGTTAACTTTA | |
|  |  | Reverse | | GGGGACCACTTTGTACAAGAAAGCTGGGTCGTTATCACGTTTACTGCTTACAGC | |
| subcellular localization | *no-spGbSBT1* | Forward | | GGGGACAAGTTTGTACAAAAAAGCAGGCTTCATGAAGGATGGTGTTTATATTGTG | |
|  |  | Reverse | | GGGGACCACTTTGTACAAGAAAGCTGGGTCGTTATCACGTTTACTGCTTACAGC | |
| Over-expression | *GbSBT1* | Forward | | GGGGACAAGTTTGTACAAAAAAGCAGGCTTCATGAAGCCCCTCGTTAACTTTA | |
|  |  | Reverse | | GGGGACCACTTTGTACAAGAAAGCTGGGTCCTAGTTATCACGTTTACTGCTTACAG | |
| BiFC | *prohibitin* | Forward | | GGGGACAAGTTTGTACAAAAAAGCAGGCTTCATGTACGATGTCCGTGGCGG | |
|  |  | Reverse | | GGGGACCACTTTGTACAAGAAAGCTGGGTCCCGGCCAACGTTCAGAAGCA | |
| qPCR | *GbSBT1* | Forward | | GGTTGATGTGTTGTCACTGTCTCTT | |
|  |  | Reverse | | GTGCTAGCAGCAACTGTCACAATC | |
| qPCR | *ubiquitin* | Forward | | CCAGAAGGAATCCACTTTGC | |
|  |  | Reverse | | CCAGCTCACATCAGCATACG | |
| qPCR | *GbPR1* | Forward | | AAGAATGTGGGTTAGTGAGAGGGT | |
|  |  | Reverse | | ACCACTTGAGTATAATGCCCGC | |
| qPCR | *GbPR2* | Forward | | TCTCGGTCCAGTCATAAACTTCTTG | |
|  |  | Reverse | | GATATGGACCATCTGTCACAACGA | |
| qPCR | *PR-2* | Forward | | GGGACGGCTCTCGTGGCTACC | |
|  |  | Reverse | | CGCGCGTTATCGAAACTCGCGG | |
| qPCR | *MAPK3* | Forward | | TGACGTTTGACCCCAACAGA | |
|  |  | Reverse | | CTGTTCCTCATCCAGAGGCTG | |
| qPCR | *MAPK6* | Forward | | CCGACAGTGCATCCTTTAGCT | |
|  |  | Reverse | | TGGGCCAATGCGTCTAAAAC | |
| qPCR | *ACT2* | Forward | | TCTTCCGCTCTTTCTTTCCAAGC | |
|  |  | Reverse | | ACCATTGTCACACACGATTGGTTG | |
| qPCR | *PDF1.2* | Forward | | TTTGCTGCTTTCGACGCAC | |
|  |  | Reverse | | CGCAAACCCCTGACCATG | |
| Yeast two hybrid | *GbSBT1* | Forward | | CGGAATTCATGAAGCCCCTCGTTAACTTTA | |
|  |  | Reverse | | ACGCGTCGACCTAGTTATCACGTTTACTGCTTACA | |
| Yeast two hybrid | *peptidyl-tRNA* | Forward | | CGGAATTCCGCGGCTACCTCATCTCCCC | |
|  | *hydrolase* | Reverse | | CGGGATCCTCACAGCAGCTTCAGATGCCC | |
| Yeast two hybrid | *prohibitin* | Forward | | CGGAATTCTACGATGTCCGTGGCGGTTC | |
|  |  | Reverse | | CGGGATCCTTACCGGCCAACGTTCAGAAG | |
| Yeast two hybrid | *benzodiazepine* | Forward | | CGGAATTCAAGTTTCCCCTCCACCCCCG | |
|  | *receptor* | Reverse | | CGGGATCCTCACAGCGCCTTGCCCTTTC | |
