# Supplementary material for: Characterization of a Novel Cotton Subtilase Gene GbSBT1 in Response to Extracellular Stimulations and Its Role in Verticillium Resistance
Source: PLoS One. 2016 Apr 18;11(4):e0153988. doi: 10.1371/journal.pone.0153988 (PMC4835097; doi:10.1371/journal.pone.0153988)
Supplement: S2 Table — (DOCX) [file pone.0153988.s005.docx]

**Table S2. The total of proteins pulled down by GbSBT1.**

| **Protein accession** | **Protein putative identity** | **Signal peptide** | **Peptide** | **Score** |
| --- | --- | --- | --- | --- |
| gi\|697086726 | peptidyl-tRNA hydrolase | Y | 13 | 378 |
| gi\|697090994 | uracil phosphoribosyltransferase | N | 19 | 283 |
| gi\|697084894 | alternative oxidase | N | 10 | 156 |
| gi\|697079755 | triosephosphate isomerase | N | 6 | 125 |
| gi\|697071457 | long-chain-fatty-acid-CoA ligase | N | 3 | 114 |
| gi\|697075038 | L-xylulose reductase | N | 7 | 113 |
| gi\|697081075 | importin alpha re-exporter | N | 6 | 111 |
| gi\|697073417 | 60S ribosomal protein L21-B | N | 8 | 106 |
| gi\|697069779 | long-chain-fatty-acid-CoA ligase | N | 6 | 96 |
| gi\|697069413 | prohibitin | Y | 3 | 95 |
| gi\|697082003 | 26S protease regulatory subunit 8 | N | 5 | 95 |
| gi\|697089827 | hypothetical protein VDAG_06823 | N | 5 | 95 |
| gi\|697069931 | pyrABCN | N | 4 | 94 |
| gi\|697067331 | hypothetical protein VDAG_04108 | N | 7 | 79 |
| gi\|697075520 | hypothetical protein VDAG_03753 | N | 2 | 76 |
| gi\|697065929 | acetyl-CoA acetyltransferase | Y | 3 | 75 |
| gi\|697070791 | T-complex protein 1 subunit alpha | N | 4 | 75 |
| gi\|697083553 | DNA mismatch repair protein | N | 2 | 75 |
| gi\|697090513 | ferrochelatase | N | 7 | 75 |
| gi\|697074896 | amidohydrolase family protein | N | 2 | 74 |
| gi\|697072427 | quinone oxidoreductase | N | 4 | 70 |
| gi\|697069241 | guanine nucleotide-binding protein alpha-2 subunit | N | 3 | 67 |
| gi\|697067657 | 26S protease subunit RPT4 | N | 4 | 66 |
| gi\|697074264 | 26S proteasome regulatory subunit RPN5 | N | 2 | 66 |
| gi\|697075309 | acetyl-CoA carboxylase | N | 4 | 64 |
| gi\|697068063 | calcium/calmodulin-dependent protein kinase | N | 2 | 61 |
| gi\|302415737 | GTP-binding protein ypt3 | N | 4 | 60 |
| gi\|697067287 | oligosaccharyltransferase alpha subunit | Y | 4 | 60 |
| gi\|697067251 | 3-ketoacyl-CoA thiolase | N | 3 | 59 |
| gi\|697067481 | carbamoyl-phosphate synthase arginine-specific large chain | Y | 3 | 59 |
| gi\|697088233 | zinc transporter SLC39A9 | Y | 4 | 59 |
| gi\|697090201 | argininosuccinate synthase | N | 3 | 59 |
| gi\|697081834 | inorganic phosphate transport protein PHO88 | N | 5 | 58 |
| gi\|697084129 | orotate phosphoribosyltransferase | N | 3 | 58 |
| gi\|697085394 | dolichol-phosphate mannosyltransferase | N | 6 | 58 |
| gi\|697070991 | CRAL/TRIO domain-containing protein | N | 4 | 57 |
| gi\|697077172 | hypoxanthine guanine phosphoribosyltransferase | N | 4 | 57 |
| gi\|302420233 | 40S ribosomal protein S24 | N | 2 | 54 |
| gi\|697067381 | uridylate kinase | Y | 4 | 54 |
| gi\|697089027 | DNA-directed RNA polymerase II subunit RPB1 | N | 4 | 54 |
| gi\|697072343 | GMP synthase | N | 4 | 53 |
| gi\|697082834 | mitochondrial 2-oxodicarboxylate carrier 1 | N | 6 | 53 |
| gi\|697070636 | cell division control protein | N | 3 | 52 |
| gi\|697079215 | NAD-specific glutamate dehydrogenase | N | 2 | 51 |
| gi\|697087482 | 26S proteasome regulatory subunit RPN9 | N | 2 | 51 |
| gi\|697089683 | 1-Cys peroxiredoxin B | N | 2 | 51 |
| gi\|697066441 | coproporphyrinogen III oxidase | N | 2 | 50 |
| gi\|697087612 | mitogen-activated protein kinase | N | 3 | 49 |
| gi\|697073758 | coatomer subunit gamma-2 | N | 2 | 48 |
| gi\|697069031 | arsenical pump-driving ATPase | N | 2 | 47 |
| gi\|697081343 | vacuolar ATP synthase 98 kDa subunit | N | 4 | 47 |
| gi\|697067669 | sterol-4-alpha-carboxylate 3-dehydrogenase | N | 2 | 46 |
| gi\|697068402 | ribulose-phosphate 3-epimerase | N | 2 | 46 |
| gi\|697068933 | hydroxymethylglutaryl-CoA synthase | N | 2 | 46 |
| gi\|302420087 | 60S ribosomal protein L24 | N | 7 | 45 |
| gi\|697072320 | hypothetical protein VDAG_07953 | N | 3 | 44 |
| gi\|697073457 | sulfate adenylyltransferase | N | 3 | 44 |
| gi\|697077246 | NADPH dehydrogenase | N | 2 | 44 |
| gi\|697080554 | armadillo/beta-catenin-like repeat-containing protein | N | 3 | 44 |
| gi\|697084086 | guanine nucleotide-binding protein alpha-3 subunit | N | 2 | 44 |
| gi\|697086240 | phosphoribosylaminoimidazole-succinocarboxamide synthase | Y | 2 | 44 |
| gi\|697068897 | 54S ribosomal protein L6 | Y | 4 | 43 |
| gi\|697086372 | malate synthase | N | 3 | 43 |
| gi\|697066557 | vacuolar protein sorting-associated protein | N | 2 | 42 |
| gi\|697071360 | ubiquinone/menaquinone biosynthesis methyltransferase ubiE | N | 3 | 42 |
| gi\|697067037 | actin-interacting protein | N | 4 | 41 |
| gi\|697084301 | vacuolar ATP synthase subunit C | N | 3 | 41 |
| gi\|697084385 | UTP-glucose-1-phosphate uridylyltransferase | N | 3 | 39 |
| gi\|697084797 | FK506-binding protein | N | 2 | 39 |
| gi\|697075719 | carbonic anhydrase | N | 2 | 38 |
| gi\|697066785 | yop-1 | N | 2 | 37 |
| gi\|697072137 | KapG | N | 2 | 37 |
| gi\|697072256 | hypothetical protein VDAG_07925 | N | 2 | 37 |
| gi\|697073862 | hypothetical protein VDAG_02599 | N | 4 | 37 |
| gi\|697090393 | importin-7 | N | 2 | 37 |
| gi\|697066901 | disulfide-isomerase erp38 | Y | 3 | 36 |
| gi\|697068973 | transport protein SEC13 | N | 2 | 36 |
| gi\|697084217 | NADH-ubiquinone oxidoreductase 29.9 kDa subunit | N | 2 | 36 |
| gi\|697087440 | coatomer subunit alpha | N | 2 | 36 |
| gi\|697066401 | pyruvate kinase | N | 4 | 35 |
| gi\|697079176 | ATP-dependent RNA helicase DBP2 | N | 2 | 35 |
| gi\|697088709 | hypothetical protein VDAG_06592 | N | 2 | 35 |
| gi\|697090329 | eukaryotic translation initiation factor 3 subunit 8 | N | 2 | 35 |
| gi\|697069673 | carbamoyl-phosphate synthase arginine-specific small chain | N | 2 | 34 |
| gi\|697084676 | calcium-transporting ATPase sarcoplasmic/endoplasmic reticulum type | N | 2 | 34 |
| gi\|697087438 | 26S proteasome regulatory subunit rpn12 | N | 2 | 32 |
| gi\|697088963 | adenine phosphoribosyltransferase | N | 3 | 32 |
| gi\|697091124 | hypothetical protein VDAG_08344 | N | 4 | 32 |
| gi\|697066123 | phosphatase PSR1 | N | 3 | 31 |
| gi\|697068358 | HNRNP arginine N-methyltransferase | N | 3 | 31 |
| gi\|697077116 | 60S ribosomal protein L13-B | N | 7 | 31 |
| gi\|697083475 | leucyl-tRNA synthetase | N | 2 | 31 |
| gi\|697088151 | transcriptional repressor rco-1 | N | 2 | 31 |
| gi\|697088689 | NAD dependent epimerase/dehydratase family protein | N | 3 | 31 |
| gi\|302404233 | histone H2B | N | 2 | 30 |
| gi\|697076302 | methylglutaconyl-CoA hydratase | N | 2 | 30 |
| gi\|697081805 | vesicular-fusion protein SEC18 | N | 2 | 30 |
| gi\|697088275 | coatomer subunit delta | N | 2 | 30 |
| gi\|697068043 | benzodiazepine receptor family protein | Y | 3 | 29 |
| gi\|697076456 | guanine nucleotide transporter | N | 3 | 29 |
| gi\|697079398 | cleavage and polyadenylation specificity factor subunit 5 | N | 2 | 29 |
| gi\|697088345 | sexual differentiation process protein isp4 | N | 2 | 29 |
| gi\|697068600 | mRNA 3~-end-processing protein RNA14 | N | 2 | 28 |
| gi\|697089965 | CTP synthase | Y | 2 | 28 |
| gi\|697090631 | voltage-gated potassium channel subunit beta-1 | N | 2 | 28 |
| gi\|697068021 | phospho-2-dehydro-3-deoxyheptonate aldolase | N | 2 | 26 |
| gi\|697072068 | nitrilotriacetate monooxygenase component A | N | 2 | 26 |
| gi\|697081813 | 40S ribosomal protein S0 | N | 2 | 26 |
| gi\|697078159 | importin subunit beta-1 | N | 2 | 25 |
| gi\|697081603 | Het-C protein | Y | 2 | 25 |
| gi\|697086394 | helicase SEN1 | N | 2 | 25 |
| gi\|697067135 | cytochrome b5 | N | 2 | 24 |
| gi\|697081591 | orotidine 5~-phosphate decarboxylase | N | 6 | 24 |
| gi\|302409274 | 60S ribosomal protein L18 | N | 9 | 23 |
| gi\|697069671 | electron transfer flavoprotein subunit beta | N | 2 | 23 |
| gi\|697070271 | cwl1 | N | 2 | 23 |
| gi\|697077958 | hypothetical protein VDAG_09803 | N | 2 | 23 |
| gi\|697070739 | coatomer subunit beta | N | 2 | 22 |
| gi\|697067213 | TATA-box-binding protein | N | 3 | 21 |
| gi\|697075994 | peroxide stress-activated histidine kinase mak2 | N | 2 | 21 |
| gi\|697068103 | helicase SWR1 | Y | 2 | 20 |
| gi\|697079644 | dTDP-D-glucose 4,6-dehydratase | N | 2 | 20 |
| gi\|697070103 | chloride channel protein | N | 2 | 18 |
| gi\|697070238 | oxidoreductase 2-nitropropane dioxygenase family | N | 2 | 18 |
| gi\|697071463 | short-chain dehydrogenase/reductase SDR | Y | 2 | 18 |
| gi\|697077881 | hypothetical protein VDAG_09776 | Y | 2 | 18 |
| gi\|697079220 | acyl-protein thioesterase | N | 6 | 18 |
| gi\|697082229 | hypothetical protein VDAG_07234 | N | 2 | 17 |
| gi\|697088955 | ATP synthase subunit g | N | 21 | 17 |
| gi\|697074911 | hypothetical protein VDAG_02970 | N | 2 | 15 |
